# *Diabrotica v. virgifera* Seems Not Affected by Entomotoxic Protease Inhibitors from Higher Fungi

**DOI:** 10.3390/insects15010060

**Published:** 2024-01-15

**Authors:** Stefan Toepfer, Szabolcs Toth, Tanja Zupan, Urban Bogataj, Nada Žnidaršič, Marta Ladanyi, Jerica Sabotič

**Affiliations:** 1 Department of Integrated Plant Protection, Plant Protection Institute, Hungarian University of Agriculture and Life Sciences (MATE), 2100 Godollo, Hungary; toth.szabolcs.1990@gmail.com; 2 CABI, 2800 Delemont, Switzerland; 3 Department of Biotechnology, Jožef Stefan Institute, 1000 Ljubljana, Slovenia; tanjazupan.10@gmail.com (T.Z.); jerica.sabotic@ijs.si (J.S.); 4 Department of Biology, Biotechnical Faculty, University of Ljubljana, 1000 Ljubljana, Slovenia; urban.bogataj@bf.uni-lj.si (U.B.); nada.znidarsic@bf.uni-lj.si (N.Ž.); 5 Department of Applied Statistics, Institute of Mathematics and Basic Science, Hungarian University of Agriculture and Life Sciences (MATE), 1118 Budapest, Hungary; ladanyi.marta@uni-mate.hu

**Keywords:** western corn rootworm, macrocypin, clitocypin, cocaprin, cospin, digestive proteases

## Abstract

**Simple Summary:**

Certain soil insects, such as the maize root-attacking larva of the western corn rootworm beetle, are increasingly difficult to control. This is because of recent bans of some insecticides. An alternative and safer approach may be the development of biological insecticides based on the insect-killing ingredients of mushrooms. We examined the effects of proteins from mushrooms that may affect the digestion of insects, called protease inhibitors. We confirmed that some of them are indeed active against digestive proteases of western corn rootworm. However, bioassays showed that they have no major effect, either on the young insect larvae, on the adult beetles, or on their eggs. This contrasts with the Colorado potato beetle, upon which some of the mushroom proteins cause mortality. Microscopic observations revealed that the structure of the gut of western corn rootworms and Colorado potato beetles are quite similar. Therefore, we suggest that the resistance of corn rootworms to those protease inhibitors might be due to effective adaptation of proteins in the gut. Despite the lack of major effects on corn rootworms, we will further study the vast variety of proteins from mushrooms, with the ultimate aim of offering alternative and biological pest management solutions.

**Abstract:**

Certain soil insects, such as the root-damaging larvae of the maize pest *Diabrotica virgifera virgifera* (Coleoptera: Chrysomelidae), are increasingly difficult to control because of recent bans of some insecticides. An alternative and safer approach may be the development of biopesticides based on entomotoxic defense proteins of higher fungi. Many of these potentially interesting proteins are protease inhibitors, and some have been shown to adversely affect insects. We examined the effects of the cysteine protease inhibitors macrocypin 1, 3, and 4 from *Macrolepiota procera*, clitocypin from *Clitocybe nebularis*, and cocaprin 1 and the serine protease inhibitor cospin 1 from *Coprinopsis cinerea* on *D. v. virgifera*. We confirmed the inhibition by mycocypins of the cysteine catalytic-type proteolytic activities in gut extracts of larvae and adults. The inhibition of *p*Glu-Phe-Leu-hydrolyzing activity was stronger than that of Z-Phe-Arg-hydrolyzing activity. Mycocypins and cospin resisted long-term proteolytic digestion, whereas cocaprin 1 was digested. Bioassays with overlaid artificial diet revealed no effects of proteins on neonatal mortality or stunting, and no effects on adult mortality. Immersion of eggs in protein solutions had little effect on egg hatching or mortality of hatching neonates. Microscopic analysis of the peritrophic matrix and apical surface of the midguts revealed the similarity between larvae of *D. v. virgifera* and the chrysomelid *Leptinotarsa decemlineata*, which are sensitive to these inhibitors. The resistance of *D. v. virgifera* to fungal protease inhibitors is likely due to effective adaptation of digestive enzyme expression to dietary protease inhibitors. We continue to study unique protein complexes of higher fungi for the development of new approaches to pest control.

## 1. Introduction

Protease inhibitors of plant origins have been studied for crop protection as they represent a natural mechanism of plants against different antagonists, including herbivorous insects [1,2,3]. Expression of protease inhibitors is often triggered when a plant is attacked. Then, these inhibitors can act in the insect gut and suppress the feeding or growth of an insect. However, during the co-evolution of plants and herbivores, insects have developed different ways to counteract the effects of protease inhibitors in their nutrition [3]. These include overexpression of digestive proteases, expression of compensatory insensitive proteases, or expression of enzymes that degrade protease inhibitors [4,5]. In contrast, proteins from mushrooms display unique characteristics that distinguish them from proteins from plants, even those with similar functionality. They are exceptionally stable proteins that can withstand extreme temperature and pH conditions, and proteolytic degradation. Furthermore, proteins from mushrooms might confer more durable defense than their plant counterparts, as herbivorous insect pests often have not co-evolved with them. Thus, it is less likely these insects have mechanisms to easily adapt to fungal proteins. Finally, the protein nature of these fungal protease inhibitors prevents them from accumulating in soils or organisms, making them suitable for development and use as biopesticides.

A few families of protease inhibitors unique to higher fungi have shown entomotoxicity, albeit to different target organisms [6]. For example, mycocypins, the cysteine protease inhibitors from *Clitocybe nebularis* (clitocypin) and from *Macrolepiota procera* (macrocypins), show activity against *Leptinotarsa decemlineata* [7]. They exhibit antinutritional effects that impair larval development when introduced into the diet as recombinant proteins or as transgenes expressed in potato. The underlying mode of action is likely the inhibition of a specific set of digestive cysteine proteases in the insect midgut. Moreover, no changes in transcription levels of known adaptation-related digestive enzymes were observed in these larval guts, as is regularly observed with other dietary cysteine protease inhibitors from various plant and animal sources, confirming their uniqueness [7,8].

Another example is the fungal serine protease inhibitors mycospins, which showed activity against *Drosophila melanogaster* [9,10]. This toxicity was mediated by specific inhibition of the fly’s serine proteases by the trypsin inhibitor cospin. Recently, protease inhibitors with dual inhibitory activity against both cysteine and aspartic proteases, termed cocaprins, have been identified in the mushroom *Coprinopsis cinerea* [11]. However, their effect against insects has not yet been investigated.

Protein digestion in the *D. v. virgifera* gut has been shown to occur predominately by cysteine proteases, with significant proteolytic activity observed in the wide range of pH 4 to pH 9 with a pH optimum between pH 5 and pH 6. In addition, studies with protease inhibitors indicate that multiple protease types are involved in protein digestion and no single inhibitor completely abolished the isolated proteolytic activity from larval guts [12]. Using specific substrates, proteolytic activities of different catalytic types were confirmed, including cysteine peptidases (cathepsin B-, L- H-like), serine peptidases (trypsin-, chymotrypsin-, elastase-, cathepsin G-, plasmin-, thrombin-like), aspartic peptidases (pepsin-like), metallopeptidases (saccharolysin-like), and aminopeptidases [13]. In addition, a great diversity of expressed cathepsin L-like and cathepsin B-like peptidases were observed, suggesting that this is a highly expressed group of proteins in the gut that play an important role in protein digestion of *Diabrotica* species [14].

Surprisingly, none of the described protease inhibitors from higher fungi seem to have been studied in detail on rootworms; or, if studied, for example by industry, results may not have been published. Rootworms are a group of soil insect pests belonging to the genus *Diabrotica* (Coleoptera: Chrysomelidae). One of the most important pests is *D v. virgifera*, which causes extensive damage to maize in North America and Europe [15]. Its three larval instars feed on maize roots and cause plant lodging. The adults feed on silk and pollen, disrupting maize pollination. *Diabrotica v. virgifera* is usually controlled by crop rotation, synthetic soil insecticides or seed coatings, entomopathogenic nematodes, or transgenic maize expressing *Bacillus thuringiensis* entomotoxic proteins. The latter is not available in some regions of the world and has also been shown to be only moderately effective against coleopterans. Recently, some of the soil insecticides or seed coatings have been banned due to high toxicity or serious non-target effects [16,17,18,19,20]. Therefore, growers are left with limited options to control such soil insect pests [15]. 

Alternatives are urgently needed, and protease inhibitors from higher fungi, as described above, may have potential. We therefore studied the effects of six dietary fungal protease inhibitors on *D. v. virgifera*, targeting different catalytic classes of proteases. Briefly, we investigated the cysteine protease inhibitors macrocypins 1, 3, and 4 from the parasol mushroom *Macrolepiota procera*, clitocypin from the clouded agaric *Clitocybe nebularis* (both Basidiomycota: Agaricaceae), as well as cocaprin 1 and trypsin inhibitor cospin from the grey shag *Coprinopsis cinerea* (Basidiomycota: Psathyrellaceae). We hypothesized that entomotoxic effects could be detected in the different life stages of *D. v. virgifera*. We primarily studied the larvae using bioassays based on artificial diet, because farmers and companies usually try to control the larvae of the pest since they cause the main damage. However, we also studied the effect of inhibitors on the adults, because their control may be warranted if egg laying and subsequent root damage the following year are significantly reduced. The egg stage of *D. v. virgifera* is not normally targeted in crop protection because the eggs are well protected and widely distributed in the soil of a field. Nevertheless, we investigated the possible effects of the inhibitors on the larvae hatching from the treated eggs, which would represent a new approach to manage this pest. We anticipated that our results may form the basis for the development of completely new types of protein-based biopesticides that use defense proteins from higher fungi. 

## 2. Materials and Methods

### 2.1. Preparation of Recombinant Protease Inhibitors for Bioassays

Recombinant fungal protease inhibitors clitocypin (Clt, UniProt ID: Q9P4A2) [21], macrocypins [22], macrocypin 1 (Mcp1, B9V973), macrocypin 3 (Mcp3, B9V979), macrocypin 4 (Mcp4, B9V982), cospin (PIC, D0EWJ0) [23], and cocaprin 1 (Ccp1, A8PCJ3) [11] were expressed using pET vectors in the bacterial expression system with *Escherichia coli* BL21(DE3). They were purified in a one-step purification protocol from inclusion bodies by solubilization in urea followed by refolding in gel filtration chromatography on a Sepharose S200 column equilibrated with Tris-HCl buffer, pH 7.5, containing 0.3 M NaCl. For cocaprin 1, an additional purification step was performed by metal affinity chromatography using TALON^®^ Metal Affinity Resin (Clontech/Takara Bio Inc., Kusatsu, Japan) according to the manufacturer’s recommendations [9,11,22,24]. The functionality of the recombinant proteins was confirmed by proteolytic assays. Clitocypin, macrocypins, and cocaprin 1 were tested against papain (EC 3.4.22.2) using benzyloxycarbonyl (Z)-Phe-Arg-7-amido-4-methylcoumarin (AMC) as substrate at pH 6.5 in 0.1 M MES buffer containing 2.5 mM DTT; for cospin, trypsin (EC 3.4.21.4) was used with the same substrate at pH 8 in 0.1 M Tris-HCl buffer containing 0.02 M CaCl_2_ and 1.5 mM EDTA, as described in [11].

### 2.2. Handling of Diabrotica v. virgifera

Eggs, larvae, and adults of *Diabrotica virgifera virgifera* (Coleoptera: Chrysomelidae, western corn rootworm, EPPO code DIABVI) were targeted. Primarily, a non-diapause colony of the insect from USDA ARS (Bookings, SD, USA) was used. Its life stages were reared under controlled conditions following the methods of [25,26,27,28,29]. 

Two weeks prior to a bioassay with eggs or larvae, soil dishes containing freshly laid eggs were removed from adult rearing cages to allow sufficient incubation time at 24 ± 2 °C in the dark until eggs hatched. Ready-to-hatch eggs were washed in clean, cool tap water containing <0.01% NaOCl and sieved through a 300 µm mesh sieve. For egg bioassays, 60 to 120 eggs were transferred from the sieve to a treatment solution (see below) using a spatula. For larval bioassays, eggs were placed in clean and moist fine sand in 15 cm diameter Petri dishes lined with wet towel paper for hatching. 

For adult bioassays, either mature adults from the non-diapausing colony or wild adults collected from heavily infested fields in southern Hungary were used. The adults (both males and females) were picked up from the rearing cages with a tube aspirator, placed in a freezer at −2 to −4 °C for 4 to 6 min, and then transferred to the wells as described below. Both the non-diapause colony and wild *D. v. virgifera* are considered susceptible to agents with new modes of action, as studied in [30,31]. 

### 2.3. Assessing the Resistance of Fungal Protease Inhibitors to Proteolytic Digestion in Gut Extracts of D. v. virgifera Larvae and Adults

The guts were dissected from untreated third instar larvae and adults of *D. v. virgifera* and homogenized in liquid nitrogen using a mortar and pestle. Crude protein extract was prepared by adding an equal volume of 25 mM MES pH 6.5 extraction buffer, vigorous mixing, and incubation on ice for 15 min, followed by removal of insoluble material by centrifugation at 4 °C and 16,000× *g* for 5 min. Protease inhibitors (6 μg at a concentration of 0.4 mg/mL) were mixed in 100 mM MES pH 6 containing 2 mM DTT with the crude protein extract from 3rd instar larval or adult guts (3 μL) that exhibited strong proteolytic activity and were incubated for 24 h at room temperature. The reactions were then stopped by boiling in SDS-PAGE loading buffer, analyzed by SDS-PAGE (15% acrylamide gel) and visualized by Coomassie blue staining.

### 2.4. Assessing Inhibition of Proteolytic Activities in Guts of D. v. virgifera Larvae and Adults

To evaluate the inhibitory potential of each recombinant protease inhibitor, they were analyzed at a concentration of 10 μM under conditions that test different catalytic protease types, as described in [8]. For detection of cysteine proteases in crude protein extracts, activity against Z-Phe-Arg-MCA, (Bachem, Bubendorf, Switzerland) and *p*Glu-Phe-Leu-*p*NA (Sigma, St. Louis, MO, USA) was measured in MES buffer, pH 6, with 5 mM DTT. For detection of serine proteases, activity against Boc-Gly-Arg-Arg-MCA (Bachem, Bubendorf, Switzerland) was measured in 100 mM Tris-HCl buffer, pH 8.8, with 5 mM EDTA and 5 mM E-64 in crude protein extracts. For detection of aspartic proteases, activity against MOCAc-Ala-Pro-Ala-Lys-Phe-Phe-Arg-Leu-Lys-(Dnp)-NH_2_ (Peptide Institute Inc., Osaka, Japan) was determined in McIlvaine citrate-phosphate buffer, pH 3.4 and pH 5.4. The inhibitors E-64 (10 μM), aprotinin (5 μM), and pepstatin A (6 μM) were used as positive controls to confirm cysteine, serine, and aspartic protease activities, respectively. The measured proteolytic activities were normalized to the negative control (control was set to 100%). Statistical methods were used to test whether the protease inhibitors could inhibit the activities in the gut extracts using a one-way ANOVA. The dependent variables were the detected proteolytic activities in crude protein extracts from the guts of third instar larvae and adults, while the factor variable was the type of protease inhibitor, such as:

- Control, PIC, E-64, Mcp1, Mcp3, Mcp4, CCP1, and Clt (tests Z-Phe-Arg-MCA and *p*Glu-Phe-Leu-*p*NA at pH 6);
- Control, pepstatin, and CCP1 (tests MOCAc-Ala-Pro-Ala-Lys-Phe-Phe-Arg-Leu-Lys-(Dnp)-NH_2_ at pH 5.4 or at pH 3.5);
- Control, aprotinin, Mcp1, Mcp3, Mcp4, and PIC (tests Boc-Gly-Arg-Arg-MCA at pH 8).

To ensure normality of the residuals, the larvae data of the Z-Phe-Arg-MCA at pH 6 were square root transformed, whilst the larvae data of *p*Glu-Phe-Leu-*p*NA at pH 6 and the adult data of Z-Phe-Arg-MCA at pH 6 were logarithm transformed. In this way, the normality of the model residuals was accepted based on the skewness and kurtosis of their distributions. Since the homogeneity of variances was still violated, we applied robust ANOVA with Welch correction. The pairwise comparisons with the control treatments were performed with one-sided Games-Howell post hoc tests, which can handle the inhomogeneity of variances.

### 2.5. Assessing Effects of Fungal Protease Inhibitors on D. v. virgifera in Bioassays

We assessed the effects of six dietary fungal protease inhibitors on *D. v. virgifera* larvae, adults, and eggs, targeting different catalytic classes of proteases (Table 1). Proteins were diluted in PBS buffer (1× phosphate-buffered saline containing 10 mM Na_2_HPO_4_, 1.8 mM KH_2_PO_4_, 137 mM NaCl, and 2.7 mM KCl, pH 7.4) or in 20 mM Tris-HCl buffer containing 0.3 M NaCl, pH 7.5, to the required concentration (Table 2). Attempts were made to standardize solutions for all experiments and *D. v. virgifera* stages, but this depended on the number of proteins available and literature information from comparable bioassay methods for comparable insect species and life stages (Table 1 and Table 2).

Positive controls used in bioassays were the neonicotinoid insecticide imidacloprid, i.e., 1-(6-chloro-3-pyridylmethyl)-N-nitroimidazolidin-2-ylideneamine [32], the cysteine protease inhibitor E-64, i.e., trans-epoxysuccinyl-l-leucylamido (4-guanidino) butane [8], and the serine protease inhibitor AEBSF, i.e., 4-(2-aminoethyl) benzenesulfonyl fluoride hydrochloride In Proceedings of the. Buffers Tris-HCl, PBS, sterile tap water, or completely untreated bioassay arenas served as negative controls.

**Table 1:** Characteristics of protease inhibitors from higher fungi tested against *Diabrotica v. virgifera* in standardized laboratory bioassays, as well as positive and negative controls. NA–not available or applicable. Families according to Merops classification (https://www.ebi.ac.uk/merops/, accessed on 10 May 2022) [33].

Treatment (Code)	Origin	Family	Molecular Weight, Size	Fold (PDB Code)	Protease Family Inhibited	Buffer	Known Entomotoxic Activity	Reference
Proteins								
Macrocypin 1 (Mcp1)	*Macrolepiota procera * (Basidiomycota: Agaricaceae)	I85	19.1 kDa 169 aa	β-trefoil (3H6Q)	Cysteine proteases: C1/C13	Tris-HCl or PBS	*Leptinotarsa decemlineata* larvae	[8,22,34]
Macrocypin 3 (Mcp3)			19.0 kDa 167 aa	β-trefoil (3H6Q)	Cysteine proteases: C1/C13	Tris-HCl		
Macrocypin 4 (Mcp4)			18.7 kDa 167 aa	β-trefoil (3H6Q)	Cysteine and serine proteases: C1/S1	Tris-HCl		
Clitocypin (Clt)	*Clitocybe nebularis * (Basidiomycota: Agaricaceae)	I48	16.7 kDa 150 aa	β-trefoil (3H6R)	Cysteine proteases: C1/C13	Tris-HCl or PBS	*Leptinotarsa decemlineata* larvae	[21,24,34,35,36]
Cocaprin 1 (Ccp1)	*Coprinopsis cinerea * (Basidiomycota: Psathyrellaceae)	I106	16.1 kDa 138 aa	β-trefoil (7ZNX)	Cysteine and aspartic proteases: C1/A1	PBS	No toxicity *Aedes aegypti* larvae	[11]
Cospin (PIC)		I66	16.7 kDa 150 aa	β-trefoil (3N0K)	Serine proteases: S1 (trypsin)	Tris-HCl	*Drosophila melanogaster* larvae	[9,37]
Positive controls								
E-64	E-64 trans-epoxysuccinyl-l-leucylamido (4-guanidino) butane		357 Da NA	epoxide (ChEMBL374508)	Cysteine proteases	DMSO	*Diabrotica undecimpunctata howardi*, *D. v. virgifera* larvae	[38,39,40,41]
AEBSF (Pefabloc SC)	4-(2-Aminoethyl) benzenesulfonyl fluoride hydrochloride)		239 Da NA	ChEMBL1096339	Serine protease	Water	*Helicoverpa armigera* gut, *Drosophila* haemocyte-like l(2)mbn cells	[23,38,42,43]
Imidacloprid	1-(6-chloro-3-pyridylmethyl)-N-nitroimidazolidin-2-ylideneamine		255.6 Da NA	ChEMBL406819	NA	Water	Broad spectrum incl against *D. v. virgifera*	[32]
Negative controls								
C_4_ H _11_ NO _3_·HCl (Tris-HCL)	Buffer (20 mM, ~7.5 pH)		NA	NA	NA	Water	NA	
Phosphate-buffered saline (PBS)	Buffer (~7.4 pH)		NA	NA	NA	Water	NA	
Water	Buffer		NA	NA	NA	Water	NA

**Table 2:** Tested concentrations of protease inhibitors from higher fungi against *Diabrotica v. virgifera* in standardized laboratory bioassays, as well as of positive and negative controls. Neonates: 96-well-plate bioassays with 20 µL overlay treatment on artificial diet-filled wells with 1 neonate per well. Adults: 6-well-plate bioassays with 40 µL overlay treatments on each artificial diet core with 3 adults per well. Eggs: Dipping assay into treatment solutions and then pipetting 20 µL treatment solution with approximately 15 ± 8 eggs on filter paper per Petri dish.

Treatment (Code)	Neonates						Adults						Eggs				
	µg/mL	µL/Arena	µg/cm^2^ *	µg/Arena	µg/Insect	µg/ 1 mg Insect	µg/mL	µL/Arena	µg/cm^2^ *	µg/Arena	µg/Insect	µg/ 1 mg Insect	µg/mL	µL/Tube	µg/Tube	µg/Insect	µg/ 1 mg Insect
Proteins																	
Macrocypin 1 (Mcp1)	1630	20	100	32.6	32.6	81.5	1630	40	28	65	22	2.2	200	200	40	0.4	20
	200	20	12	4	4	10	2400	40	41	96	32	3.2	800	200	160	1.6	80
													1630	200	326	3.3	163
													2400	200	480	4.8	240
													3200	200	640	6.4	320
Macrocypin 3 (Mcp3)	1630	20	100	32.6	32.6	81.5	1630	40	28	65	22	2.2	200	200	40	0.4	20
													400	200	80	0.8	40
													1200	200	240	2.4	120
													1630	200	326	3.3	163
													1900	200	380	3.8	190
Macrocypin 4 (Mcp4)	1630	20	100	32.6	32.6	81.5	1630	40	28	65	22	2.2	200	200	40	0.4	20
													800	200	160	1.6	80
													1630	200	326	3.3	163
													2400	200	480	4.8	240
													3200	200	640	6.4	320
Clitocypin (Clt)	1565	20	96	31.3	31.3	78.2	1565	40	27	63	21	2.1	200	200	40	0.4	20
													1410	200	282	2.8	141
Cocaprin 1 (Ccp1)	1630	20	100	32.6	32.6	81.5	1630	40	28	65	22	2.2	200	200	40	0.4	20
	200	20	12	4	4	10							800	200	160	1.6	80
													1630	200	326	3.3	163
													2400	200	480	4.8	240
													3200	200	640	6.4	320
Cospin (PIC)	1630	20	100	32.6	32.6	81.5	1630	40	28	65	22	2.2	200	200	40	0.4	20
													1630	200	326	3.3	163
Mcp1 + PIC	1630 +1360	20	100 + 100	32.6 + 32.6	32.6 + 32.6	81.5 + 81.5	1630 + 1360	40	28 + 28	65 + 65	22 + 22	2.2 + 2.2	Not tested				
Positive controls																	
E-64	163	20	10	3.3	3.3	8.2	163	40	2.8	6.5	2.2	0.2	163	200	33	0.3	16.3
AEBSF	1630	20	100	32.6	32.6	81.5	1630	40	28	65	22	2.2	200	200	40	0.4	20
													800	200	160	1.6	80
													1630	200	326	3.3	163
													2400	200	480	4.8	240
													3200	200	640	6.4	320
E-64 + AEBSF	163 +1630	20	10 + 100	3.3 + 32.6	3.3 + 32.6	8.2 + 81.5	163 + 1630	40	2.8 + 28	6.5 + 65	2.2 + 22	0.2 + 2.2	Not tested				
Imidacloprid	2	20	0.12	0.04	0.04	0.1	34	40	0.6	1.36	0.45	0.05	200	200	40	0.4	20
	3.4	20	0.2	0.07	0.07	0.17	75	40	1.3	3	1	0.1	1000	200	200	2	100
													2000	200	400	4	200
Negative controls																	
Tris-HCl	20 mM NaCl, (pH 7.5)	20						40						200			
PBS	(pH 7.4)	20						40						200			
Water		20						40						200			

* 0.34 cm^2^ top-treated diet area per well for neonates, 2.36 cm^2^ diet-core treated surface per well for adults.

#### 2.5.1. *Diabrotica v. virgifera* Larvae

To assess the effects of fungal protease inhibitors on larvae of *D. v. virgifera*, bioassays based on artificial diet were performed under controlled, semi-sterile conditions according to the methods of [17,44]. Each bioassay consisted of 6 to 7 transparent polystyrene 96-well plates (BioLite 96 well multidish, Thermo Scientific, Waltham, MA, USA). Each well had a volume of 330 µL, a diameter of 5 mm, and a height of 10 mm, giving a top surface area of 0.34 cm^2^. Each treatment was applied to a column of 8 wells on each plate. Each protein was typically tested in three experimental repetitions (true replicates). 

The larval diet for a bioassay was prepared one day before treatment and infestation. The diet was prepared under semi-sterile conditions following the methods of [17,45,46,47]. Briefly, a commercial southern corn rootworm diet was used (Frontier #F9800B, Frontier Agricultural Sciences, Newark, DE, USA), and lyophilized grinded maize roots and food coloring were added. Then, 190 µL of the diet was pipetted into each 330 µL well, filling each well approximately 2/3 full (repeater pipette P-8, Topscien Instrument Co., Ningbo, China). Plates containing the diet were then dried in a laminar flow cabinet for 45 min and then stored in a cool place at 3 to 5 °C overnight. 

The following day, 20 µL of the treatments were applied to the 0.34 cm^2^ diet surface in each well. The concentrations used are listed in Table 2. For larval bioassays, a concentration of 1630 µg protein per mL was generally prepared. This corresponds to about 33 µg of protein per arena surface per individual neonate, or about 82 μg per 1 mg of insect or 100 μg per cm^2^ of food surface. Lower or higher concentrations were used in some experiments (Table 2). The main positive control in our larval experiments was 2 or 3.4 µg imidacloprid per mL (0.01 or 0.017 μL product Confidor ™ 200 SL, Budapest, Hungary). This corresponds to 0.04 and 0.07 µg per arena and individual neonate, respectively, or 0.1 and 0.17 µg per 1 mg of organism. The order of the applied treatments was changed in every other plate to avoid edge effects. Treated plates were allowed to dry for 1 to 1.5 h followed by cooling at 3 to 5 °C for 1 h. 

A fine artist brush was used to place one neonate per well following methods of [17]. The neonates were arranged rectangularly rather than in the order of the treatment columns to avoid systemic errors. The brush was regularly cleaned in 70% ethanol followed by sterile tap water. The filled plate was sealed with an optically clear, self-adhesive qPCR sealing film (#AB-1170, Thermo Scientific), which allowed assessments without opening the plate. Four to five holes were pierced into the seal per well with flamed 00-insect pins to allow aeration. 

Plates were incubated for 5 days at 24 ± 2 °C and 50 to 70% r.h. in the dark in a ventilated incubator (Friocell 22, MMM Medcenter, Munich, Germany). 

Data were collected on days 3 and 5 of the experiments. Larval mortality, stunting, and contamination were visually assessed through the transparent seals using a stereomicroscope (10× magnification, SMZ-B4, Optec, Chongqing, China) following observation methods of [17]. On day 5, the length of surviving larvae was also measured. Data from a plate were used only if the natural mortality threshold of 37.5% was not exceeded in the untreated control, i.e., no more than 3 dead of 8 larvae per column of wells. This is in contrast to common practices in bioassays with other insects, where the quality assumption is usually <10% background mortality [48]. However, this is difficult to achieve with rootworm larvae because the artificial diets known to date can still cause some variability despite the significant recent improvements by [49].

#### 2.5.2. *Diabrotica v. virgifera* Adults

To assess the effects of fungal protease inhibitors on adult *D. v. virgifera*, artificial diet-based bioassays were performed under controlled semi-sterile conditions. Each bioassay consisted of 28 to 32 transparent polystyrene 6-well plates. The treatment wells were arranged in systematic blocks of three wells. This resulted in a total of at least 4 × 3 wells, or a minimum of 12 wells (sample size) per treatment and bioassay. Each well had a diameter of 35 mm and a height of 15 mm. Each well contained a surface-treated diet core and at least 3 beetles. This resulted in at least 36 beetles for each treatment. 

Each diet core was 7 × 5 mm, giving a treatable surface area of 71.5 mm^2^. A non-pollen, non-honey diet was adapted from [50] and prepared in boiled agar. After cooling to about 50 to 55 °C, 0.11 g of methylparaben fungicide, 3.2 mg of chlortetracycline, and 3.2 mg of streptomycin sulphate antibiotics were added per 100 mL of diet. The diet had pH 5.3 at the end of preparation. The diet was prepared semi-sterile, but autoclaving was not possible due to heat-sensitive ingredients. The diet was stored in sterile Petri dishes at 4 °C for no more than one week before use. The diet cores were prepared using a flamed iron core-cutter and transferred to each well under a laminar flow. 

Approximately 40 µL of the treatment solutions were applied over each diet core per well to achieve good coverage of the entire surface of the cores. The concentrations used are listed in Table 2. A concentration of 1630 µg per mL was typically prepared. This corresponds to approximately 65 µg per arena and 22 µg per individual adult (with three adults per well). This corresponds to approximately 2.2 µg per 1 mg of insect. Lower or higher concentrations were used in some experiments. The main positive control was 34 or 75 µg of imidacloprid per mL (0.17 or 0.375 µL of product). This corresponds to approximately 1.4 or 3 µg per arena, or 0.45 and 1 µg per adult individual, or 0.05 or 0.1 µg per 1 mg of organism. After application, the treatment dried under the laminar flow for 1 h. Mature adults were removed from a rearing cage, as described above, and placed in the wells. Plates were sealed and incubated at 24 ± 0.5 °C, 65–70% r.h., L:D 12:12.

Data were collected on days 1, 3, and 5 of the experiment, i.e., adult survival, feeding on the diet cores, sublethal effects such as abnormal leg or antenna movements, and contamination. Around 8% of wells were contaminated after 3 days of assays and 29% after 5 days. Plates were excluded from analyses if more than half of the wells were contaminated or if the food cores had dried out by day 5, which was rarely the case. 

#### 2.5.3. *Diabrotic v*. *virgifera* Eggs

To assess the effects of fungal protease inhibitors on *D. v. virgifera* eggs, filter paper bioassays were performed under controlled, semi-sterile conditions. Each bioassay consisted of 100 to 120 filter paper-filled Petri dishes with 7 dishes per treatment. The filter paper in the 5 cm diameter dishes was moistened with 100 µL of sterile tap water prior to transfer of the eggs. The sieved, ready-to-hatch-eggs were transferred from the sieve to a treatment solution in Eppendorf tubes using a fine spatula. The spatulas were flame sterilized between treatments. Approximately 60 to 100 eggs were immersed for one hour in 200 µL of treatment solution at the dosage shown in Table 2. Relatively high concentrations of µg per 1 mg of eggs were prepared. This is due to the fact that eggs are well protected against pesticides [51]. Typically, a concentration of 200 µg per mL was prepared. This corresponds to 40 µg per arena or approximately 2.7 µg per egg or 20 µg per 1 mg of organism. Lower or higher concentrations were used in some experiments (Table 2). The main positive control was 200 or 2000 µg of imidacloprid per mL (0.17 or 0.375 µL of product). This corresponds to approximately 40 or 400 µg per arena, or 2.7 or 27 µg per individual egg, or 20 or 200 µg per 1 mg of organism. After treatment, 15 ± 8 SD treated eggs were pipetted in 20 µL onto a moistened filter paper. If less than 5 eggs were transferred to filter paper, this dish was discarded and not analyzed. Then, another 100 µL of water was pipetted to distribute the eggs on the paper to facilitate data assessments. The dishes were incubated in the dark at 24 °C for one week. Data were collected under stereomicroscope on days 3, 5, and 7 of the experiments, i.e., egg hatching, mortality of newly hatched neonates, and time of hatching onset of a group of eggs in a Petri dish.

#### 2.5.4. Data Analysis

To allow comparisons between experiments, data were standardized to the data of the corresponding negative control, i.e., usually a buffer or water, as follows: Standardized data = 100 × (data in negative control–data in treatment)/maximum (data in control or in treatment). The distributions of the data were investigated using histograms and QQ normal and detrended normal probability. Skewness and kurtosis of residuals was observed for normality. Influences of treatments on neonates, adults, or eggs were analyzed using one-way ANOVA. Equality of variances was assessed using Levene’s test. Multiple comparisons were performed using the Tukey HSD post hoc comparison test for data with equal variances and the Games-Howell post hoc comparison for data with unequal variances. IBM SPSS 29 statistical software was used [52,53]. 

### 2.6. Imaging of the Midgut Structure of Larvae

Microscopic analysis of the *D. v. virgifera* midgut was performed at the level of light and electron microscopy, focusing on the interface between the midgut cells and midgut lumen. The ultrastructure of peritrophic matrix (PM) and apical surface of the midgut cells was determined. The 3rd instar larvae of *D. v. virgifera* from rearing cages were anesthetized on ice and their midguts were dissected with fine forceps and precise surgical scissors. Dissection was performed in a droplet of 0.1 M HEPES buffer. The isolated midguts were fixed in 2.5% glutaraldehyde and 2% formaldehyde in 0.1 M HEPES buffer, rinsed, and then post-fixed in 1% OsO_4_ in the same buffer. After rinsing in buffer, samples were dehydrated in graded ethanol series and acetone and embedded in blocks of Agar 100 epoxy resin. Sectioning was performed on Reichert Ultracut S (Leica, Vienna, Austria) ultramicrotome to obtain semi-thin (0.5 μm) and ultrathin (70 nm) sections. Semi-thin sections were mounted on glass slides, stained with Azure II–methylene blue, and examined using the AxioImager Z.1 (Zeiss, Jena, Germany) light microscope, equipped with the AxioCam HRc (Zeiss) camera and AxioVision (Zeiss) software. Ultrathin sections were picked on copper mesh grids, contrasted with uranyl acetate or UA Zero solution (Agar Scientific, Cambridge, UK) and lead citrate, and imaged with the CM100 transmission electron microscope (Philips, Eindhoven, The Netherlands) equipped with Orius SC200 and Bioscan (Gatan Inc., Washington, DC, USA) cameras and Digital Micrograph Suite (Gatan) software. To compare the midgut structure of *D. v. virgifera* with another coleopteran plant pest, the midguts of the third and fourth instar larvae of the Colorado potato beetle (*Leptinotarsa decemlineata,* Coleoptera: Chrysomelidae) were prepared and imaged using the same procedure.

## 3. Results

### 3.1. Inhibition of Proteolytic Activities in Guts of D. v. virgifera Larvae and Adults

**Figure 1:**
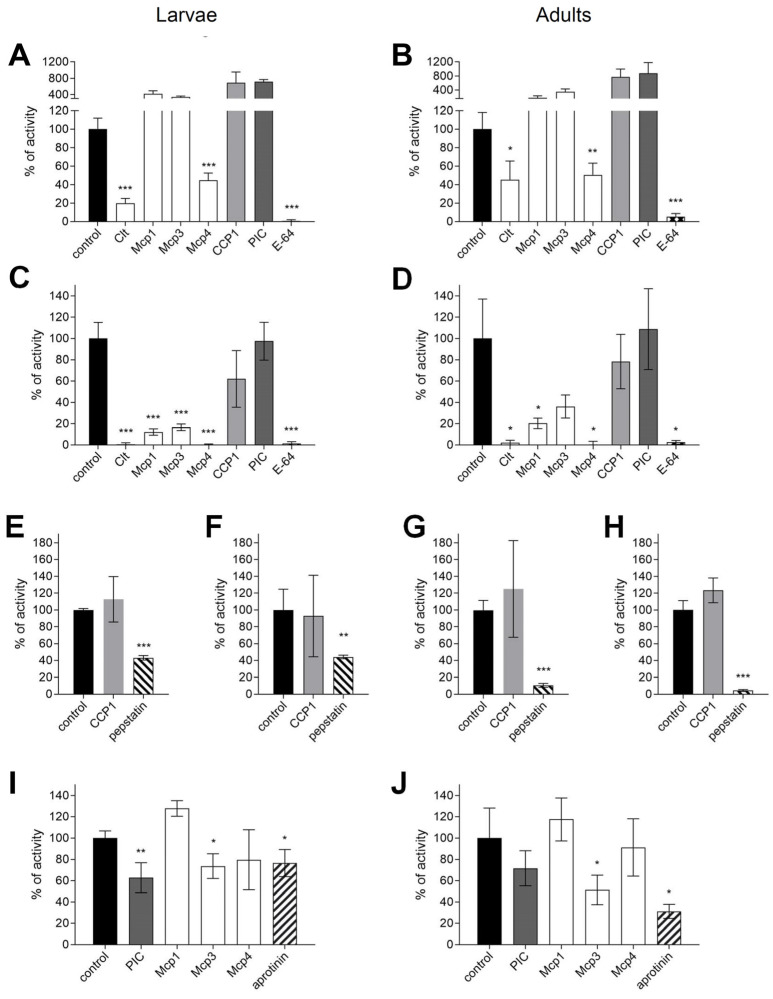
Analysis of inhibition of proteolytic activities in gut extracts of *Diabrotica v. virgifera* larvae and adults. Proteolytic activities and inhibition by fungal protease inhibitors at 10 μM and other indicated protease inhibitors were analyzed in crude protein extracts from the guts of third instar larvae (**A**,**C**,**E**,**F**,**I**) and adults (**B**,**D**,**G**,**H**,**J**). Cysteine protease activity was determined at pH 6 with Z-Phe-Arg-MCA (**A**,**B**) and *p*Glu-Phe-Leu-*p*NA (**C**,**D**) substrates using E-64 as a class-specific inhibitor to confirm cysteine protease activity. Aspartic protease activity was determined at pH 3.5 (**E**,**G**) and pH 5.4 (**F**,**H**) with the substrate MOCAc-Ala-Pro-Ala-Lys-Phe-Phe-Arg-Leu-Lys(Dnp)-NH_2_ using pepstatin A as a class-specific inhibitor. Serine protease activity was determined at pH 8.8 (**I**,**J**) in the presence of E-64 using the substrate Boc-Gly-Arg-Arg-MCA and aprotinin. Mean and standard deviation of activity measurements (%) are shown. The negative control was set to 100% and the activity measurements under different treatments were adjusted to the control. Comparisons with the control were performed by one-sided Games-Howell post hoc tests at significance levels of *** *p* < 0.001, ** *p* < 0.01, and * *p* < 0.05. The ANOVA results were all significant ((**A**–**D**): F_(df1=7; df2>16.5)_ > 31.4, *p* < 0.001; (**E**–**H**): F_(df1=2; df2>5.95)_ > 16.9, *p* < 0.01; (**I**,**J**): F_(df1=5; df2>13.19)_ > 26.3, *p* < 0.001).

Proteolytic activity of the cysteine, serine, and aspartic type was, as described above, detected in the crude protein extract of guts dissected from 3rd instar larvae and from adults (Figure 1). To evaluate the potential of those fungal protease inhibitors to control *D. v. virgifera*, the inhibition of their detected proteolytic activities was investigated. Cysteine peptidase activity against Z-Phe-Arg-MCA at pH 6 (Figure 1A,B) representing cathepsin-L- and cathepsin-B-like activities was inhibited by clitocypin (Clt) and macrocypin 4 (Mcp4). Clt showed greater inhibition of larval extracts than of adults. Mcp4 showed a comparable extent of inhibition in larvae and adults. Interestingly, some protease inhibitors (Mcp1, Mcp3, CCP1, PIC) appear to increase Z-Phe-Arg-cleaving proteolytic activity in extracts from larval and adult guts. Complete inhibition by E-64 confirmed that cysteine protease activity was indeed measured. In addition, activity against *p*Glu-Phe-Leu-*p*NA substrate was analyzed at pH 6 (Figure 1C,D), as this activity has been shown to cleave protease inhibitors cystatins as part of the adaptation response to plant inhibitors in larvae of *L. decemlineata* [8,54]. All cysteine peptidase inhibitors (mycocypins and cocaprins) inhibited larval *p*Glu-Phe-Leu-cleaving activity in *D. v. virgifera*, although mycocypins were more potent. Only mycocypins, and not cocaprins, inhibited adult *p*Glu-Phe-Leu-cleaving activity. As expected, cospin (PIC) showed no inhibition and E-64 showed complete inhibition of *p*Glu-Phe-Leu hydrolyzing activity. In addition, cocaprin 1 showed no inhibition of aspartic protease activity using the substrate MOCAc-Ala-Pro-Ala-Lys-Phe-Phe-Arg-Leu-Lys-(Dnp)-NH_2_ at pH 5.4 (Figure 1F,H) or at pH 3.5 (Figure 1E,G). For serine proteases, the substrate Boc-Gly-Arg-Arg-MCA was tested at pH 8.8 in the presence of E-64 (5 μM) because this substrate can also be cleaved by cysteine proteases (Figure 1I,J). Weak inhibition by PIC and macrocypin 3 (Mcp3) was observed in larval extracts, whereas only weak inhibition by Mcp3 was observed in gut extracts from adults. The inhibition profiles of proteolytic activities in the gut suggested that fungal protease inhibitors have the potential to affect the growth of *D. v. virgifera* through inhibition of digestive proteases in larvae and adults.

### 3.2. Effects of Fungal Protease Inhibitors on D. v. virgifera Larvae, Adults and Eggs

**Figure 2:**
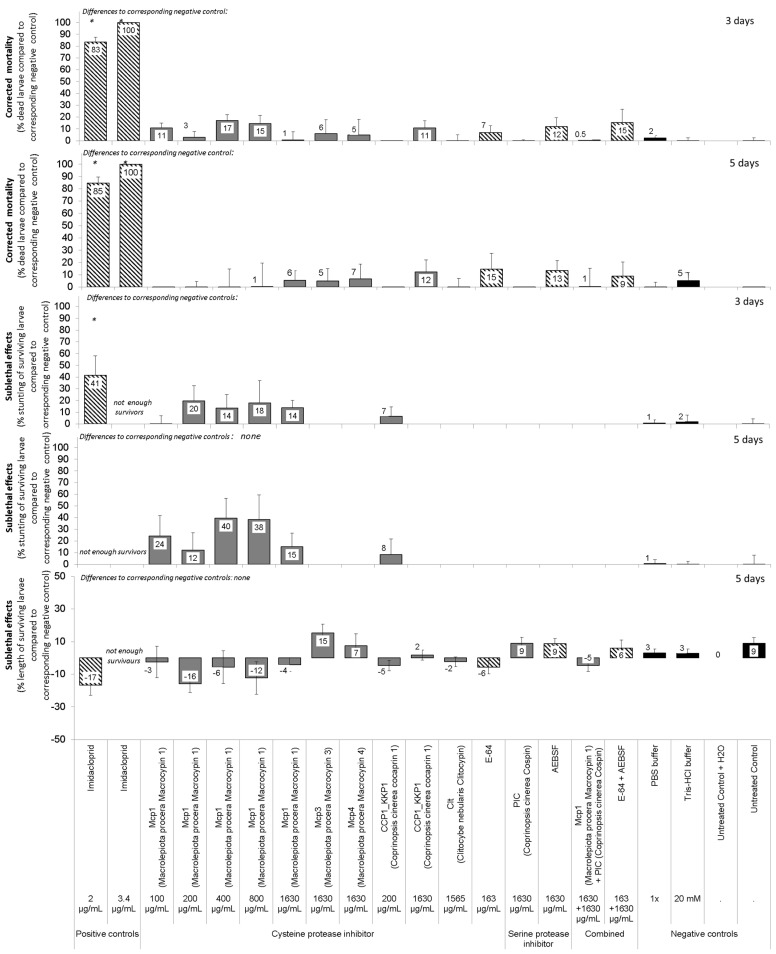
Relative effects of cysteine or serine protease inhibitors from higher fungi on neonate larvae of *Diabrotica v. virgifera.* Six to seven 96-well plates with 8 wells /treatment, total 48 wells and larvae/treatment/each of the 3 to 4 experiments. Artificial diet-based bioassay with 20 µL overlay treatments on diet. Asterisks on bars indicate significant differences between a treatment and the corresponding control according to the Games-Howell post hoc test at *p* < 0.05 following one-way ANOVA. Error bars are standard errors of the mean.

The six fungal protease inhibitors tested did not cause mortality or stunting in neonate *D. v. virgifera* at the concentrations used (Table 3, Figure 2). Treatments generally had a slight effect on neonate growth as measured by insect length, but relations were weak (see low R^2^ in Table 3). Post hoc multiple comparison tests failed to detect such effects for any particular treatment. Treatment with a mixture of cysteine protease inhibitor (Mcp1) and serine protease inhibitor (PIC) showed no synergy. The positive control, i.e., the insecticide imidacloprid, caused rapid mortality and some stunting. E-64 and AEBSF were not effective against neonates at the concentrations tested.

**Figure 3:**
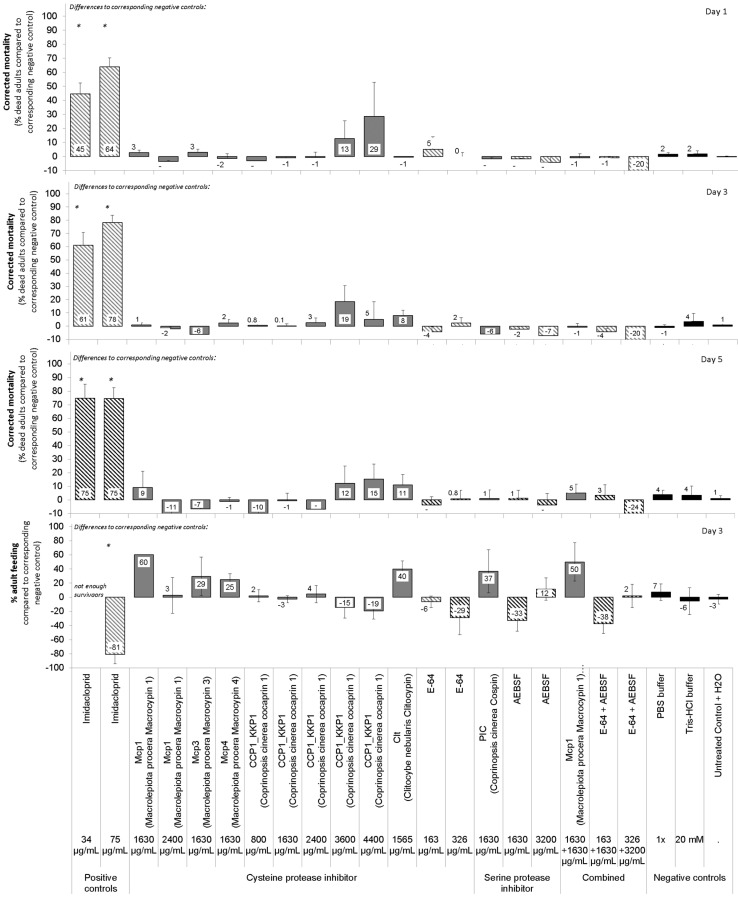
Relative effects of fungal protease inhibitors from higher fungi on adult *Diabrotica v. virgifera*. Twelve wells, i.e., 36 adults/treatment/3 to 4 experiments each. Artificial diet bioassay with 40 µL overlay treatments on each diet core. Asterisks on bars indicate significant differences between a treatment and the corresponding negative control according to the Games-Howell post hoc test at *p* < 0.05 following one-way ANOVA. Error bars are standard errors of the mean.

The fungal protease inhibitors tested did not cause major mortality, sublethal effects, or feeding inhibition in adult *D. v. virgifera* at the concentrations used (Table 2 and Table 3, Figure 3). Nevertheless, a minor treatment effect of these protease inhibitors on adult mortality was observed (Table 3). Post hoc multiple comparison tests failed to detect such effects for any particular protein treatment. The positive control, i.e., the insecticide imidacloprid, caused rapid mortality and prevented some beetle feeding. E-64 and AEBSF were not effective against adults at the concentrations tested.

**Figure 4:**
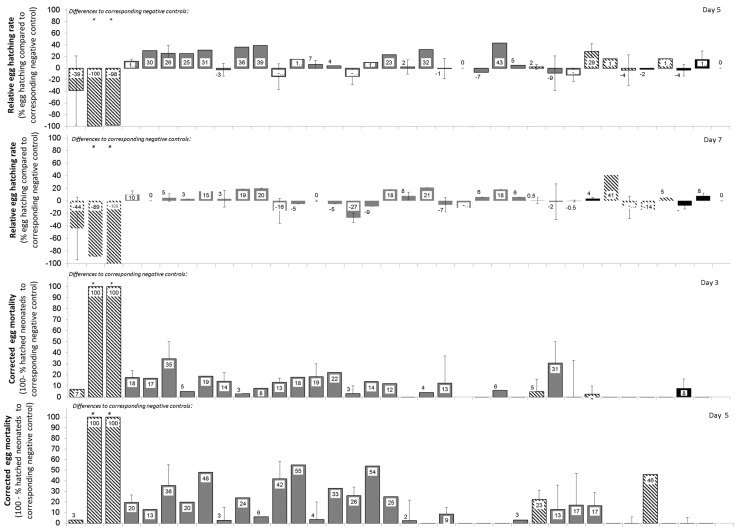

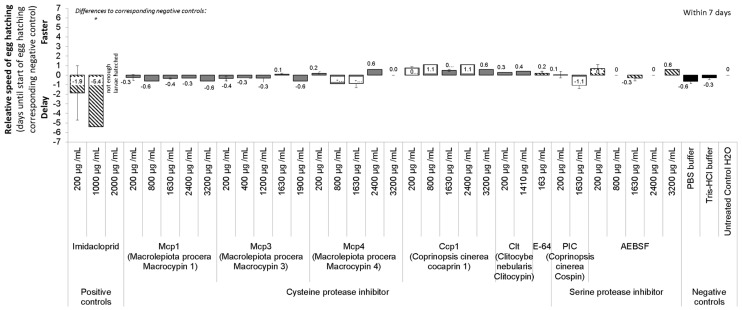
Relative effects of fungal protease inhibitors from higher fungi on eggs and egg hatching of *Diabrotica v. virgifera*. Failure in eggs to hatch indicates egg mortality. Eggs were immersed into 200 µL treatment and then 20 µL transferred to moist filter paper in 5 cm Petri dishes. Seven Petri dishes with 15 ± 8 ready-to-hatch eggs/treatment/3 to 4 experiments each. Asterisks on bars indicate significant differences between a treatment and corresponding negative control according to the Games-Howell post hoc test at *p* < 0.05 following one-way ANOVA. Error bars are standard errors of the mean.

The fungal protease inhibitors tested did not cause major mortality of eggs or newly hatched neonates or any major delay in egg hatching at the concentrations used (Table 2 and Table 3, Figure 4). Nevertheless, a minor treatment effect of these protease inhibitors on timing of start of egg hatching was observed (Table 3). However, post Hoc multiple comparison tests failed to detect such effects for any particular protein treatment. The positive control, i.e., the insecticide imidacloprid, caused some mortality of eggs and hatching larvae and a slight delay in hatching of eggs at the high concentrations of 1000 or 2000 μg/mL. E-64 and AEBSF were not effective against eggs at the concentrations used.

### 3.3. Resistance of Fungal Protease Inhibitors to Proteolytic Digestion in Gut Extracts of D. v. virgifera Larvae and Adults

**Figure 5:**
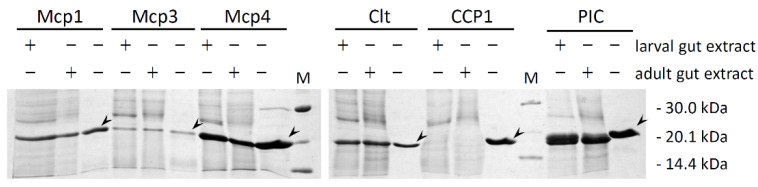
Proteolytic digestion of fungal protease inhibitors by proteolytic activities in the gut extract of *Diabrotica v. virgifera* larvae and adults. Protease inhibitors were incubated with crude protein extracts of larvae or adults as indicated and analyzed by SDS-PAGE after 24-h incubation at room temperature and visualized by Coomassie staining. Protease inhibitors are indicated by arrows in each control lane and molecular mass markers by M above the lane. All protease inhibitors were added to the reactions in the same amount (6 μg).

To assess the nature of a potential adaptative response in the *D. v. virgifera* guts, we investigated whether these protease inhibitors are degraded or resist degradation by proteolytic activities in the larval or adult gut. After 24 h of incubation at room temperature, we found that CCP1 was digested by gut proteases, whereas Clt, Mcp1, Mcp3, Mcp4, and PIC resisted proteolytic digestion (Figure 5). Further analysis showed that CCP1 was completely digested in the larval or adult gut extract after four hours of incubation at room temperature.

### 3.4. Structural Features of Midguts 

**Figure 6:**
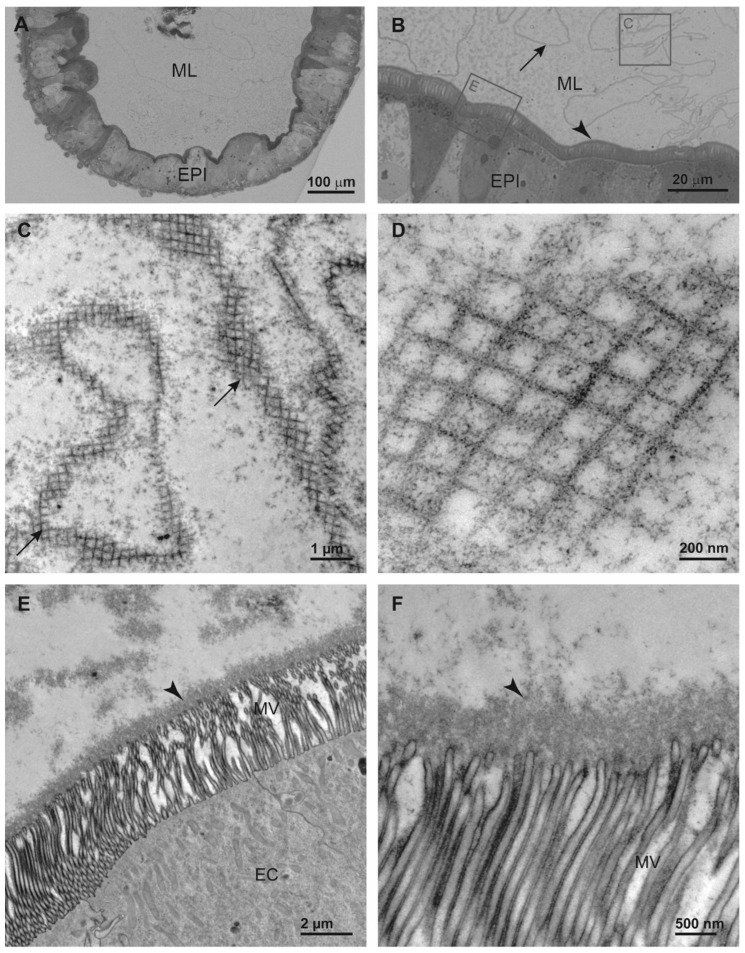
Midgut structure of larvae of *Diabrotica v. virgifera*. Columnar midgut epithelium (EPI) with numerous enterocytes characterized by long microvilli on the apical surface and several profiles of peritrophic matrix (arrows) visible in the midgut lumen (ML) in the light microscopy images (**A**,**B**). Positions of panels C and E are indicated by the squares in panel B. Ultrastructure of the peritrophic matrix organized in an orthogonal network as revealed in ultrathin sections by transmission electron microscopy (**C**,**D**). Ultrastructure of the apical surface of enterocytes (EC) (**E**,**F**) dominated by microvilli (MV) and prominent amorphous coating (arrowhead) that is continuous and covers the entire microvillar surface.

**Figure 7:**
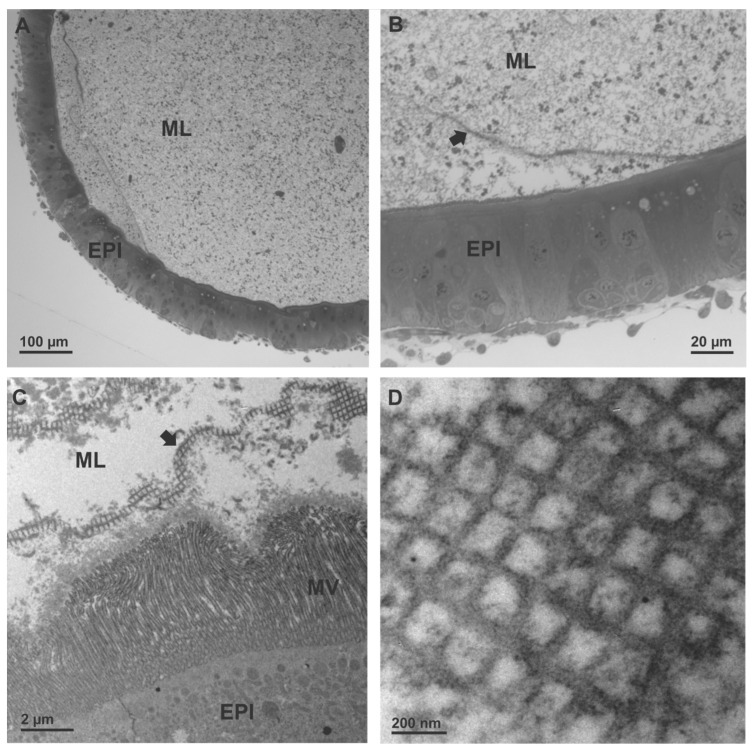
Midgut structure of larvae of *Leptinotarsa decemlineata*. Transverse sections of the midgut imaged by light microscopy (**A**,**B**) show the columnar epithelium (EPI), which comprises several cell types and faces the midgut lumen (ML) apically and hemocoel basally. Sheets of the peritrophic matrix (arrow) that compartmentalize the midgut lumen are discerned. Transmission electron microscopy imaging reveals the ultrastructure of the peritrophic matrix (**C**,**D**) as an orthogonal meshwork above the cells and a prominent brush border of microvilli (MV) on the apical surface of the midgut enterocytes.

As the epithelium of the digestive tract is positioned at the interface between the external and internal environment of an animal and functions as an absorptive and protective surface, we examined the structure of key selective barriers in the midgut. This is the peritrophic matrix that separates midgut cells from food and the apical surface of midgut cells. The midgut epithelium of 3rd instar larvae of *D. v. virgifera* comprises several cell types with numerous absorptive enterocytes characterized by long microvilli that increase the surface area of the apical cell membrane (Figure 6). The scaffold of the peritrophic matrix (PM) in *D. v. virgifera* larvae is an orthogonal network. In addition to the orthogonal PM network, a thick layer of amorphous material was found to directly cover the entire surface of midgut cells. The thickness of this electron dense coating is approximately 0.6 μm, constituting a continuous layer on the apical surface overlaying numerous microvilli of midgut cells. The general midgut structure of *D. v. virgifera* larvae is similar to that of *L. decemlineata* larvae and both display an orthogonal PM meshwork with similar ultrastructure (Figure 7).

## 4. Discussion

Recent bans on some soil insecticides and insecticidal seed coatings [16,17,18,19,20] have led to an urgent demand for novel solutions. Certain soil-dwelling insects such as wireworms (Coleoptera: Elateridae), grubs (Coleoptera: Melolonthidae or Scarabeidae), or rootworms (Coleoptera: Chrysomelidae) are becoming increasingly difficult to control. Therefore, alternative and safer approaches are urgently needed, preferably with novel modes of action. Options include biopesticides based on entomopathogenic fungi or bacteria [56], which are, however, often difficult and costly to register as microbial plant protection products. Biopesticides based on single active ingredients such as entomotoxic proteins may be the more promising approach. However, finding such novel active ingredients has proven difficult, despite high-throughput screenings by industry [44]. 

Another possibility is to develop biopesticides based on proteins from higher fungi that have a defense function against insects. Some of these are protease inhibitors, and they have been shown to adversely affect some insects [8,23,36]. Moreover, these inhibitors from higher fungi are resistant to environmental extremes such as temperature or pH, making them suitable candidates for climate-smart pest management [6]. 

We therefore investigated the inhibition of proteolytic activities in *D. v. virgifera* larval and adult guts to determine whether protease inhibitors from higher fungi have the potential to inhibit digestive proteolytic activity in one of the major maize pests, *D. v. virgifera*. The predominant cysteine protease activity was investigated using two different substrates. This showed that the cathepsin-L- and cathepsin-B-like Z-Phe-Arg-MCA hydrolyzing proteases were partially inhibited by the mycocypins Mcp4 and Clt, but not by other inhibitors tested, which, on the contrary, actually enhanced this type of proteolytic activity. Enhancement of proteolytic activity by fungal proteins with beta-trefoil fold such as mycocypins has already been observed for the serine protease trypsin and the cysteine protease papain [57]. On the other hand, *p*Glu-Phe-Leu-*p*NA hydrolysing cysteine proteases were strongly inhibited by all the mycocypins tested, i.e., Clt, Mcp1, Mcp3, and Mcp4. This type of activity was also the predominant proteolytic activity at pH 6.5 in the midgut of larvae of *L. decemlineata*, which was inhibited by macrocypins and weakly by clitocypin [8,36]. Cocaprin 1 showed weak inhibition of *p*Glu-Phe-Leu-*p*NA activity in larval guts but not in adults. Moreover, aspartic proteolytic activity was not inhibited by cocaprin 1, suggesting that cocaprins target specific aspartic proteases, since fungal rhizopuspepsin was also not inhibited, and varying degrees of inhibition were observed for the animal aspartic proteases pepsin, rennin, and aspartic proteases of the nematode *Haemonchus contortus* [11]. Interestingly, the activity of chymotrypsin-like serine proteases detected with Boc-Gly-Arg-Arg-MCA was only weakly affected by macrocypin 3, as well as by cospin in larvae, but only by macrocypin 3 in adults. In contrast, this type of activity was not inhibited by macrocypins or clitocypin in extracts from the guts of larvae of the *L. decemlineata*, which is a chrysomelid such as *Diabrotica* spp. [8,36]. The differences in the inhibitory profiles of digestive proteases between the *Leptinotarsa* and *Diabrotica* spp. indicate that, despite their similarity in a cysteine protease predominance, their spectrum of intestinal proteases differs markedly. This might be due to differences in their diet. Nevertheless, the observed inhibition of digestive proteases of the cysteine catalytic type, which is the predominant digestive proteolytic activity in *D. v. virgifera,* was promising for testing the potential of fungal protease inhibitors to control *D. v. virgifera*.

We therefore evaluated the fungal protease inhibitors for their potential effects on eggs, larvae, and adults of one of the most important maize pests, *D. v. virgifera*. Unfortunately, none of the cysteine protease inhibitors tested showed any appreciable activity against the different life stages of *D. v. virgifera*, i.e., not macrocypins 1, 3, and 4, clitocypin, cocaprin 1, or cospin. This is surprising, as these proteins have been relatively well studied for their protease inhibitory effects on different organisms, including nematodes [58], larvae of *L. decemlineata* [8,36], and *D. melanogaster* [9]. Even the larvae of *Diabrotica undecimpunctata* howardi or *D. v. virgifera* were shown to be affected at least by the cysteine protease inhibitor E-64 [38,39,40,59], which could not be confirmed in our study. Also, the standard serine protease inhibitor AEBSF (Pefabloc) is known to affect the *Helicoverpa armigera* gut or the haemocyte-like l(2)mbn cells of *Drosophila* spp. [23,43], but did not appear to have any major effect on *D. v. virgifera* in our study. It could therefore not be used as a positive control in experiments with *D. v. virgifera*, as originally planned.

It could be argued that plant protection effects do not necessarily represent mortality, but that sublethal, repellent, or growth-influencing effects may play a greater role. We attempted to address this question by examining treated insects for numerous types of sublethal effects in addition to mortality. These included possible stunting or feeding inhibition in larvae, delay in hatching or deformation of newly hatching neonates from eggs or their inability to fully exit the eggshell, or sublethal effects such as agitated movements of legs or antennae in adults. However, our bioassays with overlaid artificial diet showed no major sublethal effects by the tested proteins. 

It may also be argued that the concentrations used of inhibitors may have been too low [60]. However, we based our choice of concentration (Table 2) on the few available and comparable studies. For example, the ED_50_ of Cry3 protein from *Bacillus thuringiensis*, which is toxic to rootworm, is reported to be roughly 1 µg protein/cm^2^ diet surface, and about 2 µg is often used per cm^2^ in standard bioassays with 96-well plates, which corresponds to about 34 µg protein per mL [61]. We used a double to nearly 10-fold higher concentration against neonates in our study to ensure not to miss any potential minor effects. Unfortunately, the dosages used in published studies are often difficult to compare with each other, as often only information per mL is provided but the amount of the applied agent per arena or per insect is missing, or vice versa. Ref. [60] tested 50 µg of potato cysteine proteinase inhibitor-10 (PCPI-10) per mL in 14-well plates against first instar *Diabrotica undecimpunctata* larvae, and [62] tested 250 µg soybean cysteine proteinase inhibitor (scN soyacystatin) per mL, and 2000 µg cysteine proteinase inhibitor E-64 per mL against first instar *Diabrotica undecimpunctata* in 24-well plates. Ref. [39] estimated an ED_50_ of 25 µg and an ED_70_ of 31 µg of potato multicystatin (PMC) per cm^2^ against first instar *Diabrotica undecimpunctata* in 24-well plates. Finally, ref. [63] reported an ED_50_ of 53 µg PIP47A toxin from *Pseudomonas mosselii* per mL against first instars of several *Diabrotica*, including *D. v. virgifera* which relates to 0.53 µg per neonate and to 1.3 µg per 1 mg insect. We used 10 to 82 µg per 1 mg insect (Table 2). When it comes to adult or egg assays for insects using protease inhibitors, the dosage information for standardized laboratory experiments is scarce. We have attempted to convert the dosage information from larval bioassays to dosages potentially required for adults and eggs as a function of mg body weight. Therefore, we believe that we have tested sufficient concentrations of inhibitors against the different life stages of *D. v. virgifera* in our study. However, we cannot completely exclude the possibility that some effects on the insect, especially on the well-protected eggs, would be observed at higher concentrations. 

Another aspect is that the protease inhibitors studied here appear to be relatively specific for certain organisms, although only a limited number of organisms have been studied so far. For example, the cysteine protease inhibitors Mcp 1 and 4 have been shown to affect the growth of *L. decemlineata* larvae [8], but not *D. melanogaster.* The effect of Mcp3 on insects has not yet been demonstrated, but Mcp3 has been shown to have weak nematotoxicity against *Caenorhabditis elegans* [58]. No major effects on larval or adult *D. v. virgifera* growth or development was detected in our study. Clitocypin is known to affect growth of *L. decemlineata* larvae [36], but not nematodes [58] or *D. melanogaster* [10,23]. Finally, the studied serine protease inhibitor cospin 1 is known to affect the development of *D. melanogaster,* but not *L. decemlineata* larvae and not *D. v. virgifera*, as reported here. Thus, the observed differences in the inhibition profiles of the fungal protease inhibitors might prevent their effect on *D. v. virgifera*. The surprising resistance of *D. v. virgifera* to dietary protease inhibitors may be possibly due to effective adaptation processes in the expression of digestive proteases [64], or structural characteristics of the gut may prevent the interaction of inhibitors with digestive proteases in the gut. 

Because the peritrophic matrix (PM) and apical surface of epithelial cells are the two main selective barriers in the insect midgut that impede passage of xenobiotics from the food into tissues, we examined their ultrastructure in *D. v. virgifera* larvae in comparison to *L. decemlineata* larvae, which had previously been shown to be sensitive to some fungal protease inhibitors, as described above. The orthogonal architecture of the PM in *D. v. virgifera* larvae, as revealed by transmission electron microscopy of midgut ultrathin sections, is similar to that previously reported in several insects [65] and it also resembles the PM in *D. undecimpunctata* [66] and the PM in *L. decemlineata* larvae (Figure 6 and Figure 7). In addition to the PM, a thick, continuous coating was conspicuous in *D. v. virgifera* larvae, covering the entire microvillar surface and providing an additional protective structure between the gut lumen and cells. This coating is not organized in a characteristic PM orthogonal network but consists of amorphous electron dense material that forms a thick, homogeneous sheet without any distinct sublayers. Furthermore, this coating is not formed in between the microvilli, as has been reported in the literature for the synthesis of new orthogonal PMs [67]. A general model of new PM formation in insects suggests that chitin synthesis produces a chitinous meshwork of PM between the microvilli that is subsequently delaminated. The thick continuous layer covering the entire microvillar surface observed in our study of *D. v. virgifera* larvae is similar to the intestinal epithelial glycocalyx, a glycoprotein-rich protective layer that has been extensively studied and thoroughly characterized in the mammalian intestine and is suggested to also function as a deformable size-exclusion filter [57,68,69]. Thus, our results did not reveal any remarkably distinct ultrastructural features of the PM or apical surface of midgut cells in *D. v. virgifera* larvae from rearing culture compared to *L. decemlineata* larvae. However, it must be emphasized that the permeability of the PM and its ability to neutralize the effects of dietary xenobiotics depend on both PM structure and PM composition, the latter of which was not examined here and only limited data for beetles has been reported.

To determine whether one of the adaptation strategies is digestion of dietary protease inhibitors, we tested whether fungal protease inhibitors resist degradation by proteolytic activities in the guts of *D. v. virgifera* larvae or adults. This is because these protease inhibitors have been previously shown to resist digestion by proteinase K [70]. As well, mycocypins have been shown to resist digestion by adaptive proteases that cleave plant cysteine protease inhibitors in the gut of *L. decemlineata* [8,36]. In our study, clitocypin, macrocypins 1, 3 and 4, and cospin also resisted digestion by gut proteases of *D. v. virgifera* for 24 h, whereas cocaprin 1 was digested after 4 h. These results suggest that, with the exception of cocaprin 1, resistance to fungal protease inhibitors in the diet is due either to overexpression of digestive proteases that are inhibited by protease inhibitors or to expression of insensitive proteases that overcome the presence of protease inhibitors in the diet. Moreover, the composition of digestive proteases differs between these two organisms, as reflected in their inhibition profile. However, the few observed inconsistent trends showing higher mortality and/or sublethal effects on certain days or in certain bioassays (see variability in Figure 2, Figure 3 and Figure 4) only support the suggestion of successful adaptation processes. Interestingly, the adaptation of *D. v. virgifera* larvae seems more effective than that of *L. decemlineata* larvae, in which significant growth delay and reduction in weight gain due to these fungal protease inhibitors were observed [8,36]. It is possible that *D. v. virgifera* larvae come into contact with fungal hyphae and their fungal protease inhibitors due to their subterranean habitat, whereupon they may develop a more efficient adaptation to their presence than leaf-feeding larvae of *L. decemlineata*, which are less likely to come into contact with fungi.

## 5. Conclusions

In conclusion, despite their confirmed inhibition of digestive proteases, the cysteine, serine, or aspartic protease inhibitors from higher fungi studied here do not appear to have major effects on the development or survival of the different life stages of *D. v. virgifera*, most likely due to their effective adaptation to dietary protease inhibitors. Nevertheless, we will continue to explore the diversity of unique protein complexes of higher fungi, many of which are still unknown and some of which may offer new approaches to pest management.

## Figures and Tables

**Table 3:** Effects of cysteine or serine protease inhibitors from higher fungi on life stages of *Diabrotica v. virgifera*. Data were standardized relative to corresponding negative controls and pooled from at least 3 experiments for neonates, adults, and eggs, respectively. Neonates: 96-well-plate bioassays with 20 µL overlay treatment on artificial diet-filled wells, degrees of freedom 20; 245 to 341; Adults: 6-well-plate bioassays with 40 µL overlay treatments on each artificial diet core, df 21; 234; Eggs: 7 dishes of 20 µL treatment solution with approximately 15 ± 8 eggs on filter paper per treatment, df 32; 64. Positive controls were removed from the general linearized model. n.s. not significant, * significant at *p* < 0.05. False discovery rate corrected for all parameters assessed using [55].

Treatment Effects	Adjusted *R*^2^	F	*p*	fdr-Corrected *P*	Significance
on neonates					
Mortality within 3 days	0.05	1.9	0.29	0.29	n.s.
Mortality within 5 days	0.03	1.4	0.11	0.18	n.s.
Stunting within 3 days	0.01	1.2	0.29	0.29	n.s.
Stunting within 5 days	0.04	1.6	0.06	0.15	n.s.
Length within 5 days	0.09	2.6	0.0003	0.002	*
on adults					
Mortality within 1 day	0.07	1.9	0.011	0.022	*
Mortality within 3 days	0.08	1.9	0.007	0.022	*
Mortality within 5 days	0.01	1.1	0.311	0.31	n.s.
Feeding within 3 days	0.03	1.3	0.173	0.23	n.s.
on eggs					
Egg hatching rate within 5 days ^1^	−0.14	0.8	0.78	0.78	n.s.
Egg hatching rate within 7 days ^1^	−0.01	0.9	0.52	0.78	n.s.
Mortality of neonates hatched around day 3	−0.06	0.9	0.64	0.78	n.s.
Mortality of neonates hatched around day 5	0.24	1.6	0.08	0.2	n.s.
Delay in egg hatching	0.43	2.5	0.005	0.025	*

^1^ Failure in egg hatching reflects egg mortality.

## Data Availability

Descriptive summary data statistics are available at R Studio Shiny under https://tszfreeac.shinyapps.io/funcontrapestshinyapp_v2023/ (accessed on 10 May 2022).
